# Potential application of nanodelivery systems targeting ELAVL1 in prostate cancer treatment

**DOI:** 10.3389/fonc.2025.1609712

**Published:** 2025-09-01

**Authors:** Shasha Min, Mengmeng Guo, Jianuo Du, Jiaohuang Chen, Yanting Shen, Fuwen Yuan, Zhong Wang, Zhonglin Cai

**Affiliations:** ^1^ School of Gongli Hospital Medical Technology, University of Shanghai for Science and Technology, Shanghai, China; ^2^ Department of Urology and Andrology, Shanghai Pudong New Area Gongli Hospital, Shanghai, China; ^3^ School of Integrative Medicine, Shanghai University of Traditional Chinese Medicine, Shanghai, China

**Keywords:** prostate cancer, nanodelivery system, ELAVL1 (HuR), siRNA, cancer

## Abstract

**Introduction:**

Prostate cancer (PCa) is one of the most common malignant tumors in men, with increasing incidence and mortality rates, and its treatment still faces many challenges and unmet needs. ELAVL1 (human antigen R, HuR) is an RNA-binding protein that plays a crucial role in the development and progression of various cancers. Studies have shown that ELAVL1 is highly expressed in PCa and that inhibiting its expression significantly reduces prostate cell proliferation and metastasis. However, the clinical application of ELAVL1-targeting therapies remains limited by the lack of effective delivery strategies. In this context, recent advances in nanodelivery systems offer promising solutions, providing both enhanced targeting efficiency and insights for future prostate cancer treatment strategies.

**Objective:**

This review aims to explore the potential of ELAVL1-targeted therapy based on nanodelivery systems in PCa, analyze its advantages and challenges, and provide insights into future research directions.

**Methods:**

A systematic review of recent literature summarizing the expression characteristics and biological functions of ELAVL1 in PCa was conducted. Additionally, the advantages, challenges, and applications of various nanomaterials in cancer therapy are discussed.

**Results:**

Nanodelivery systems have shown significant potential in the treatment of prostate cancer.

## 1 Prostate cancer

### 1.1 Epidemiology

Prostate cancer (PCa) is one of the most common malignancies in men and presents a range of treatment challenges. It is the most prevalent malignant tumor of the male urinary and reproductive system worldwide (1). Currently, approximately 10 million men are diagnosed with PCa globally, with approximately 700,000 cases being metastatic (2). In 2022, 1.5 million new cases of PCa were reported, accounting for 7.3% of all cancer cases. Among the most common cancers in men, PCa has the second highest incidence after lung cancer, with a rate of 14.2%. A total of 397,000 global deaths from PCa were reported, corresponding to a mortality rate of 7.3% (3). According to statistics from health research centers in Europe and the United States, in 2024, 299,000 new cases of PCa and 35,250 related deaths were estimated to occur in the United States alone (4). The incidence and mortality of PCa are closely linked to age (3). The average age at diagnosis is approximately 65 years, with early symptoms, including increased urinary frequency, nocturia, and difficulty urinating. Among men aged 65 years and older, the prevalence of PCa is approximately 60% (5). Although the incidence of PCa in China is much lower than that in Western countries, a gradual increase in cases has been observed in recent years (6). This trend is likely related to improved living standards, a greater awareness of health care, widespread health screens, and the extensive use of prostate-specific antigen (PSA) testing (7).

### 1.2 Pathogenesis

The pathogenesis of PCa (PCa) is complex and involves multiple genetic and environmental factors. The development of PCa is closely linked to the accumulation of somatic mutations in the genome of prostate epithelial cells (5). Mutations in the BRCA1 and BRCA2 genes significantly increase the risk of PCa (8), whereas mutations in the HOXB13 gene are associated with familial PCa. In primary PCa, the most commonly recurrently mutated genes include TP53 (17%), SPOP (8%), AR (7%), FOXA1 (7%), and PTEN (6%) (9). FOXA1 is reported to be the third most frequently mutated gene in PCa (10). As FOXA1 is a suppressor of neuroendocrine differentiation, the loss of FOXA1 expression can promote the progression of neuroendocrine prostate cancer (NEPC) (10). FOXA1 function is altered by both coding and noncoding mutations, which may contribute to the development of PCa (11). Additionally, c-MYC is one of the key drivers of the onset and progression of PCa (12). The c-MYC gene is often amplified and upregulated in PCa, and its increased expression correlates with disease progression and castration-resistant prostate cancer (CRPC) (13). Abnormalities in DNA damage repair (DDR) pathways are also a significant mechanism involved in the development of PCa. DDR defects are widespread in PCa, with common genomic alterations such as TMPRSS2-ERG translocations, SPOP mutations, and deletions of PTEN or CHD1, all of which are closely associated with impaired DDR phenotypes (14).

The androgen receptor (AR) pathway is widely considered to play a central role in the initiation and progression of PCa. In 1941, Huggins and Hodges first reported the importance of androgen signaling in PCa, showing that orchiectomy could induce tumor regression (15). Androgen deprivation therapy (ADT), which lowers serum androgen levels and inhibits AR activity, is typically the first-line treatment for PCa. However, patient responses to ADT are heterogeneous, with 20–30% of cases progressing to CRPC (4). AR expression is nearly ubiquitous in both primary and metastatic PCa (16), and abnormal activation, mutation, or overexpression of AR are considered critical drivers of PCa initiation and progression (17). Mutations and amplifications in the AR gene are observed in approximately 1% of patients with primary PCa (18) and approximately 60% of patients with metastatic PCa (19). Patients with metastatic PCa who are treated with AR antagonists exhibit a higher mutation rate than those receiving only ADT (20). These mutations can convert AR antagonists into AR agonists, thus promoting cancer progression (21). Furthermore, these mutations allow other adrenal-derived androgens, such as progesterone, dehydroepiandrosterone, and androstenediol, to activate AR (22–25), which may explain the development of resistance to castration therapy in tumors harboring AR mutations. FOXA1 is a key regulator of the AR signaling pathway, and its mutation can affect the interaction between FOXA1 and AR, altering the androgen signaling that drives both normal prostate growth and PCa cell survival (26). The PI3K pathway is another critical oncogenic signaling pathway in PCa (27). This pathway is often aberrantly activated in PCa, promoting cell proliferation and survival. Inflammatory cytokines, such as CCR9, IL-6, and TLR3, participate in the apoptosis of PCa cells by modulating the PI3K/AKT signaling pathway. Additionally, the PI3K/AKT pathway is closely associated with mechanisms involving androgen, 1α,25-dihydroxyvitamin D3 (1α,25(OH)_2_D_3_), and prostaglandins and is regulated by ErbB, EGFR, and the HER family (28). The PIK3R1 gene, which has been identified as a tumor suppressor, encodes the PI3K subunit p85α, which acts by regulating and stabilizing p110α (29). Studies have shown that PIK3R1 can be directly suppressed by androgens in PCa (30), suggesting that PIK3R1 may be a potential biomarker for PCa prognosis and progression (27). ADT and AR inhibitors are frontline treatments for highly aggressive PCa (31). However, prolonged AR inhibition can trigger the compensatory activation of the PI3K pathway, typically due to the genomic loss of the tumor suppressor PTEN, which accelerates disease progression to the CRPC stage (32). Nikhil et al. (33) elucidated a novel mechanism of PTEN downregulation triggered by LIMK2. LIMK2 is a CRPC-specific target, and inhibiting LIMK2 can maintain the activity and stability of PTEN, thereby preventing progression to CRPC and the development of ADT resistance. The Tribbles (TRIB) protein family, consisting of TRIB1, TRIB2, and TRIB3, has been shown to participate in cancer-related processes (34) and plays a regulatory role in activating oncogenic signaling pathways such as the MAPK and PI3K-AKT pathways (35). The TRIB1 gene is located on chromosome 8q24.13, near c-MYC, and is amplified in cancer. Shahrouzi et al. (36) observed that TRIB1 is the most highly expressed gene in the c-MYC amplification locus in PCa and that its aberrant expression is associated with the pathogenesis of PCa. Furthermore, NFATc1 has been identified as a crucial molecule involved in the development of PCa. The overexpression of NFATc1 significantly promotes PCa cell growth, proliferation, and metastasis via the regulation of multiple signaling pathways (37). NFATc1 expression is upregulated through pathways such as the ERK1/2/P28/MAPK, PTEN/AKT, CaN/NFAT, and RANKL pathways. Given its pivotal role in PCa progression, NFATc1 has significant potential as an effective target for the clinical treatment and prevention of PCa metastasis (12).

ELAVL1 is an important RNA-binding protein that has been shown to be highly expressed in various cancers, including lung cancer, liver cancer, and pancreatic cancer, where it significantly promotes tumorigenesis and progression (38, 39). Additionally, ELAVL1 is associated with chemotherapy resistance (40) and radioresistance (41), highlighting its role in the treatment response. Recently, ELAVL1 was identified as one of the m6A regulatory factors that functions as a “reader” by binding to RNAs carrying m6A modification sites, thereby increasing RNA stability (41). In cancer research, ELAVL1 has also been reported to interact with molecules such as YTHDC1 and IGF2BP1 to synergistically stabilize RNA (42). Previous studies have shown that ELAVL1 is highly expressed in PCa and contributes to tumorigenesis and progression (43). Silencing ELAVL1 significantly inhibits PCa cell proliferation and promotes apoptosis, suggesting that ELAVL1 acts as an oncogene in PCa (44). According to data from TCGA database, ELAVL1 expression is higher in most PCa samples than in adjacent normal tissues, and its expression increases with an increasing Gleason score, indicating that elevated ELAVL1 expression is closely associated with tumor progression in PCa (45). Our preliminary research revealed that ELAVL1 interacts with the RNA and proteins of several m6A-binding proteins (46), suggesting that ELAVL1 may act as an upstream regulatory molecule in the m6A modification process. By modulating the expression of various downstream m6A regulators, ELAVL1 likely influences the m6A modification process. Therefore, ELAVL1 is considered an important target molecule in PCa. In addition to genetic factors, environmental and lifestyle factors, such as dietary habits, obesity, smoking, and alcohol consumption, also increase the risk of PCa (47). Chronic prostatitis and infections within the prostate may further increase the cancer risk. Inflammation is thought to contribute to cancer development by inducing cell proliferation, causing DNA damage, and promoting the formation of a tumor microenvironment.

### 1.3 Current diagnostic and therapeutic challenges in PCa treatment

**Figure 1:**
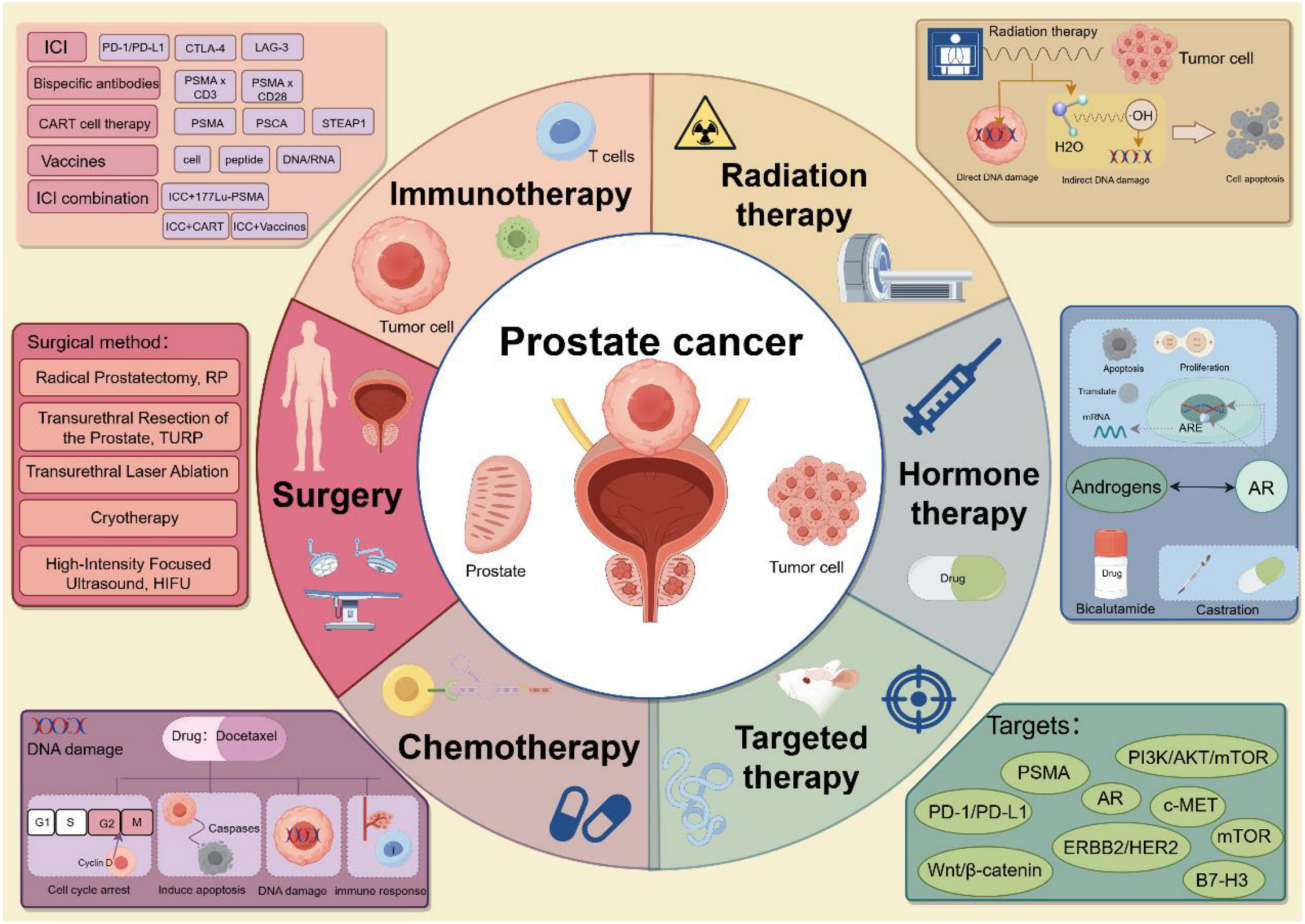
Prostate cancer treatment approaches. The above pictures are drawn by Figdraw.

Early-stage PCa often presents without obvious clinical symptoms. However, as a tumor progresses to malignancy, patients may begin to experience symptoms such as increased nocturia, urinary difficulties, and frequent urination. While various clinical treatment options are available for PCa (**Figure 1**), the mortality rate remains high. Over the past decade, the mortality rate for PCa has declined (4), yet the etiology of the disease remains poorly understood, and its pathogenesis is highly complex (3). Among PCa cases, adenocarcinomas originating from the acini tend to have a better prognosis than those originating from the ducts (5). PCa exhibits high tumor heterogeneity, with significant variations in mortality and incidence rates (5), and different tumor cells harbor distinct genetic mutations and phenotypic characteristics. Multiple tumor foci within the prostate may exhibit genetic differences, further contributing to varying degrees of metastatic spread and treatment resistance (48). This heterogeneity complicates treatment, as it also affects patient responses to therapy and the development of drug resistance. Currently, the early detection of PCa relies primarily on serum prostate-specific antigen (PSA) levels, but considerable controversy exists surrounding the use of PSA as a screening marker (48). PSA lacks sufficient specificity for PCa, as elevated PSA levels can also result from conditions such as benign prostatic hyperplasia (BPH) and prostatitis (49). PCa patients with distant metastases generally have a poor prognosis, with a five-year survival rate of only 30% (4). Bone metastasis is the most common site of distant spread, and it is the leading cause of death in PCa patients (49). Approximately 5–10% of patients who are newly diagnosed with PCa present with bone metastasis (50). Androgen deprivation therapy (ADT) is the primary treatment for bone-metastatic PCa; however, long-term ADT often leads to the development of resistance. Amplification, mutation, and splice variants of the androgen receptor (AR) are believed to contribute to this resistance (51). Studies have shown that within 1–3 years of ADT, metastatic castration-sensitive PCa inevitably progresses to metastatic castration-resistant PCa (49). Currently, early diagnostic tools are insufficiently precise, making the identification of high-risk patients in a timely manner challenging. Additionally, dormant cells within the tumor microenvironment may contribute to relapse after treatment (52).

## 2 Role of ELAVL1 in PCa

### 2.1 Molecular function of ELAVL1

Embryonic lethal abnormal vision-like protein 1 (ELAVL1), also known as HuR, is a ubiquitously expressed RNA-binding protein that plays a crucial role in posttranscriptional regulation by influencing the stability of target mRNAs (53). Under normal conditions, ELAVL1 is predominantly localized in the nucleus, but it can translocate to the cytoplasm in response to specific stimuli, where it performs its primary function—stabilizing mRNA and regulating its expression (54). ELAVL1 is a member of the ELAVL protein family, which includes HuR, HuB, HuC, and HuD. Among these family members, HuR is the only member that is widely expressed across all human tissues, whereas the other family members are expressed primarily in neuronal cells. The function of HuR depends on its three RNA recognition motifs (RRMs), which allow it to specifically bind target mRNAs (55) and modulate gene expression at the posttranscriptional level by either inhibiting degradation or promoting translation (56).

### 2.2 Role of ELAVL1 in cancer

ELAVL1 is closely linked to the development and progression of various inflammatory diseases, metabolic disorders, and cancers (57). Numerous studies have shown that ELAVL1 is overexpressed in a range of cancers, including lung, liver, and pancreatic cancers, where it promotes tumorigenesis by stabilizing the mRNAs of cancer-related genes. Additionally, ELAVL1 has been implicated in the development of resistance to cancer therapies in several malignancies, including PCa (58), pancreatic cancer (59), oral cancer (60), and colorectal cancer (61). Its role in drug resistance suggests that ELAVL1 may serve as a novel therapeutic target, as well as a critical biomarker for evaluating treatment efficacy and prognosis.

### 2.3 ELAVL1 in PCa

#### 2.3.1 Expression of ELAVL1 and its association with PCa

Studies have shown that in normal prostate epithelial cells, ELAVL1 is expressed at low to moderate levels in the nucleus (43). However, in PCa cells, ELAVL1 expression is significantly upregulated in both the cytoplasm and nucleus. Immunohistochemical staining revealed that the intensity of ELAVL1 staining in PCa tissues is markedly higher than that in adjacent nontumor tissues (46). Further investigation of the transcriptomic differences between high-ELAVL1 and low-ELAVL1 PCa cases indicated that high-ELAVL1 PCa is enriched with genes involved in RNA metabolism. Overall, ELAVL1 is overexpressed in PCa tissues, and silencing ELAVL1 significantly inhibits PCa cell proliferation while promoting apoptosis, suggesting that ELAVL1 acts as an oncogene in PCa (43).

#### 2.3.2 ELAVL1 as an RNA-binding protein that regulates mRNA and circRNA stability in PCa

Recent research has highlighted the critical biological role of circular RNAs (circRNAs) in PCa progression. For example, circFOXO3 (62), circ005276 (63) and circAMOTL1L (64) have been implicated in regulating gene expression through transcriptional control. One such circRNA, circDDIT4, forms through backsplicing of exon 2 in the linear DDIT4 mRNA, and its high expression significantly inhibits PCa cell proliferation while inducing apoptosis. In contrast, the mutated form of circDDIT4 (circDDIT4-mut) loses these effects (44). ELAVL1 was identified as a key RNA-binding protein that interacts with circDDIT4. ELAVL1 typically binds to AU-rich elements (AREs) in the 3’ untranslated region (UTR) of target genes, thereby stabilizing RNA and prolonging the half-life of mRNA (65). Anoctamin 7 (ANO7), a gene that is highly expressed in prostate epithelial cells, is considered an important prognostic marker for aggressive PCa (66). Research has shown that circDDIT4 and ELAVL1 regulate the expression of ANO7, with ANO7 overexpression promoting PCa cell proliferation and migration. ANO7 silencing partially reverses the oncogenic effects of circDDIT4 knockdown, emphasizing the role of ANO7 in the circDDIT4-ELAVL1 axis. By competitively binding to ELAVL1 via its 3’ UTR, circDDIT4 acts as a protein sponge, reducing the expression of ANO7 (44), thus promoting PCa cell apoptosis and inhibiting cell proliferation and metastasis. In conclusion, ELAVL1 functions as an oncogene in PCa, promoting cell proliferation and inhibiting apoptosis, whereas circDDIT4 suppresses its oncogenic effects by sequestering ELAVL1. This interaction significantly impacts the expression and stability of downstream target genes, thereby regulating PCa progression.

#### 2.3.3 Role of ELAVL1 in regulating the m6A modification in PCa

As an m6A-modifying factor, ELAVL1 can bind to mRNAs that are modified with m6A, increasing their stability (67). Studies have shown that ELAVL1 interacts with various m6A-binding proteins, such as YTHDC1 and IGF2BP1 (42, 68), to collectively promote RNA stability. A disruption of the m6A modification is associated with tumorigenesis and cancer progression (69). The expression of several m6A regulatory molecules, including METTL3, FTO, ALKBH5, and YTHDF3, has been shown to significantly alter tumor progression by affecting cell proliferation, migration, and invasion.

ELAVL1 interacts with multiple m6A-binding proteins at both the RNA and protein levels (46), suggesting that ELAVL1 might function as an upstream regulator of the RNA m6A modification. It is capable of influencing the expression of various m6A regulatory factors, thereby impacting m6A modifications. Consequently, ELAVL1 is considered a critical therapeutic target in PCa. Our previous transcriptomic analyses comparing high-ELAVL1-expressing PCa with low-ELAVL1-expressing PCa revealed the significant enrichment of RNA metabolism-related genes and altered expression of m6A modification factors in tumors with high ELAVL1 levels. These findings suggest that ELAVL1 may regulate RNA stability and drive cancer progression through m6A modifications (46). Moreover, METTL3 expression is closely associated with ELAVL1, indicating that ELAVL1 might further promote PCa progression by regulating METTL3 (46).

PD-L1, a well-established immune checkpoint molecule, contains m6A sites and has been implicated in various tumors, where its expression is regulated by m6A. Studies have shown that PCa with high ELAVL1 expression exhibits immunosuppressive properties (45). Knocking down ELAVL1 reduces PD-L1 expression and m6A levels in PCa. ELAVL1 interacts directly with the PD-L1 mRNA and increases its stability via m6A modifications, thereby suppressing CD4^+^ T-cell infiltration and leading to immune evasion (45).

**Figure 2:**
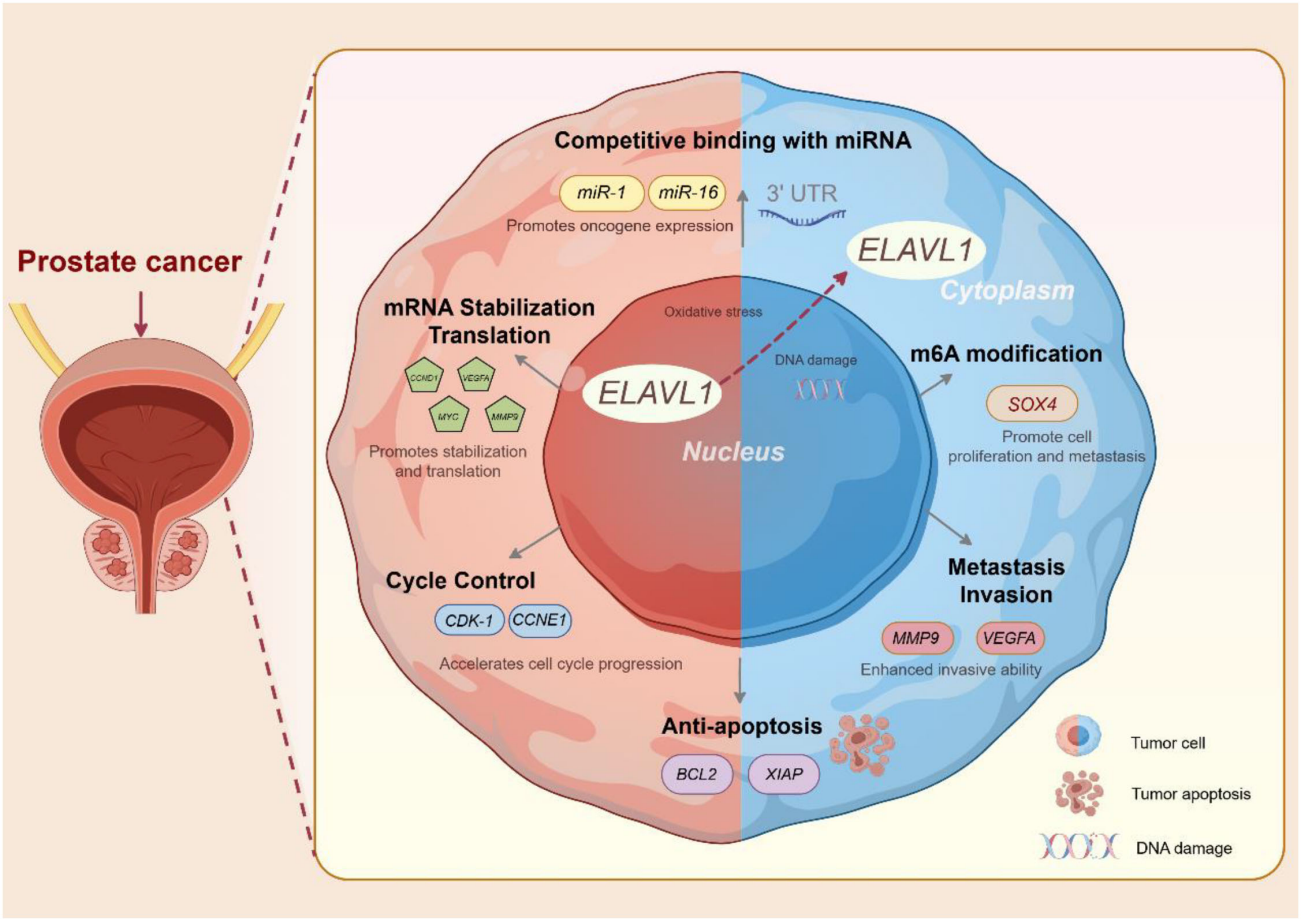
Molecular regulatory mechanism of ELAVL1. The above pictures are drawn by Figdraw.

In summary, ELAVL1 is a key oncogene in PCa that promotes tumor proliferation and metastasis while inhibiting apoptosis through multiple mechanisms, including stabilizing mRNAs and circRNAs and regulating m6A modifications. Further investigations into the molecular regulatory functions of ELAVL1 are promising for the development of novel therapeutic strategies for PCa **(** **Figure 2** ***).***

## 3 Nanoparticle delivery systems

Nanoparticle drug delivery systems are solid colloidal particles with diameters ranging from 10 to 1,000 nm that are capable of encapsulating or modifying drugs within their core or on their surface (70). In recent years, nanocarrier-mediated drug delivery systems (NDDSs) have shown significant potential in prostate cancer therapy. NDDSs utilize nanomaterials to transport drugs directly to tumor sites, promoting drug accumulation in diseased areas and enhancing therapeutic efficacy (71).

### 3.1 Targeting mechanisms and nanomaterials in NDDSs

Drug delivery via NDDSs can be classified into passive targeting and active targeting (72). Passive targeting relies on the enhanced permeability and retention (EPR) effect of tumor tissues, as well as characteristic changes in the tumor microenvironment, such as hypoxia and an acidic pH. These features facilitate the accumulation of nanocarriers at the tumor site, thereby improving the antitumor effects of drugs and reducing systemic side effects (73). However, active targeting exploits the metabolic or structural differences between tumor cells and normal cells. This activity is achieved by functionalizing nanocarriers with ligands that specifically recognize and bind to tumor cells. Common ligands include small molecules, peptides, biotin, and aptamers (72). In prostate cancer, prominent active targets include prostate-specific membrane antigen (PSMA), folate receptors, CD13, and CD14, among others (74).

The materials commonly used in nanoparticle delivery systems are summarized in the following table (**Table 1**).

**Table 1:** Advantages and disadvantages of different types of nanomaterials.

Material type	Advantage	Disadvantage	Typical materials	Mode of action
1. Polymer nanomaterials	Good biocompatibility, mature preparation technology, biodegradable (75)	Need to ensure drug stability and controlled release	Polylactic acid ethanol (PLGA), chitosan (76)	BIND-014: Polyethylene glycol–polylactic acid forms a hydrophilic shell that targets PSMA glutaric acid derivatives. Promotes the binding of prostate cancer cells to BIND-014 (77).
2. Metal nanomaterials	Photothermal effect, photodynamic effect, radiosensitization (78)	The toxicity of metal element aggregation requires evaluation in long-term clinical trials (78).	Gold nanoparticles, iron oxide nanoparticles	PSMA-targeted docetaxel-loaded superparamagnetic iron oxide nanoparticles for the treatment of prostate cancer (79).
3. Lipid nanomaterials	Bioabsorbable, strong barrier penetration, and prolonged drug circulation time (80)	The drug loading and release efficiency need to be optimized (80).	Phospholipids, cholesterol	Improve hydrophilicity through PEGylation and prolong the time of liposomal drugs in the blood circulation (81).
4. Carbon nanomaterials	Good biocompatibility, cell absorption, high surface activity (82)	May cause an immune response and toxicity	Carbon nanotubes, graphene, graphene oxide	The SWCNT-DOX targeted delivery system uses polysaccharides (sodium alginate and CS) to control the release of DOX, and FA is used to improve the targeting performance of carbon nanotubes (83).
5. Ceramic nanomaterials	Good biocompatibility, cell absorption, high surface activity (84)	The production cost is high and further research on biocompatibility is needed.	Mesoporous silica, calcium phosphate, hydroxyapatite	Green synthesis of CuCo2O_4_/CuO ceramic nanocomposites using *Dactylopius coccus* for antitumor effects through sonochemically assisted thermal decomposition (85).

Nanodelivery systems offer several key advantages.

a. Through surface modification and functionalization, nanoparticles enhance cellular interactions and uptake efficiency (86). b. These systems prolong the drug circulation time *in vivo*, allowing for controlled release via polymer degradation. This property reduces the damage caused by chemotherapeutic agents to normal tissues (87). c. The nanoscale size of particles facilitates enhanced drug accumulation at tumor sites via the enhanced permeability and retention (EPR) effect, thereby improving therapeutic efficacy (88). d. By incorporating chemical groups or targeting ligands onto their surface, nanoparticles can achieve specific responsiveness or active targeting capabilities (89). Most importantly, nanodelivery systems significantly improve therapeutic outcomes while minimizing the side effects of chemotherapeutic drugs, making them an ideal tool for anticancer treatments (90).

### 3.2 Feasibility of nanocarriers for ELAVL1 siRNA delivery

Small interfering RNA (siRNA) is a double-stranded RNA molecule composed of 21–23 nucleotides that specifically suppresses the expression of target genes (91). Synthetic siRNAs designed to target specific genes are widely applied in various biomedical fields, including the development of gene-targeted therapies (92). By leveraging the RNA interference (RNAi) mechanism, siRNAs can inhibit the expression of cancer-associated genes and mRNAs with high specificity, preventing the production of disease-driving proteins (93). In cancer research, siRNA technology has been used in both *in vitro* cell models and *in vivo* preclinical studies to identify critical molecules involved in cancer progression (94). ELAVL1 is highly expressed in prostate cancer and contributes to tumor progression, chemoresistance (40), and radioresistance (41). siRNA-mediated suppression of ELAVL1 effectively inhibits prostate cancer cell growth and viability, positioning siRNA as a powerful therapeutic tool because of its high specificity and tunability. For example, prostate cancer progression to castration-resistant stages is linked to changes in the expression of specific genes, such as AKR1C3 and AR-V7. These genes promote the development of castration-resistant prostate cancer (CRPC) by catalyzing androgen synthesis or sustaining AR signaling in an androgen-deprived environment. Chen et al. (95) utilized mesoporous silica nanoparticles to deliver an AKR1C3 siRNA, effectively reducing AKR1C3 expression and intracellular androgen levels in CRPC cell lines (C4-2, 22RV1, and VCaP) and animal models. This siRNA blocked AR signaling activation and suppressed CRPC progression.

Similarly, loading an ELAVL1 siRNA into nanodelivery systems enables precise targeting and efficient gene silencing. Muralidharan et al. (96) developed HuR siRNA-loaded nanoparticles and observed their efficacy in H1299 and CCD16 cell lines, where treated cells exhibited significantly reduced proliferation and HuR expression. These findings underscore the therapeutic potential of HuR siRNAs in cancer treatment.

Compared to traditional delivery systems such as lipid nanoparticles (LNPs), viral vectors, and antibody-drug conjugates (ADCs), ELAVL1-specific nanocarriers offer a more functionally tailored approach based on post-transcriptional regulation (97). While LNPs have shown great success in siRNA delivery, they rely heavily on passive targeting and are prone to accumulation in off-target organs like the liver (98). In contrast, ELAVL1-targeting systems can achieve tumor-specific delivery by exploiting the overexpression and cytoplasmic translocation of ELAVL1 in prostate cancer cells. ELAVL1 siRNA systems can intervene at the RNA level, enabling broader and more upstream modulation of oncogenic signaling. However, ELAVL1 nanodelivery systems currently lag behind in clinical development maturity and manufacturing scalability, indicating a need for further optimization and comparative preclinical validation.

## 4 Advantages and challenges of nanodelivery systems

Nanocarrier-based ELAVL1 siRNA delivery systems significantly increase siRNA stability and targeting efficiency, thereby improving gene silencing outcomes. The primary advantages of nanocarriers include the following: a. protection and stability—nanocarriers shield siRNAs from degradation by nucleases, thereby extending their half-life *in vivo (* 99); b. targeting capability—surface modifications enable targeted delivery to specific cells or tissues, improving the cellular uptake efficiency (100); c. reduced off-target effects—by enhancing siRNA delivery to target cells, nanocarriers minimize off-target effects and associated side effects (101); and d. controlled release—Nanocarriers allow for the sustained release of siRNAs, maintaining prolonged gene silencing effects. Various nanocarrier designs, including liposomes, polymer nanoparticles, and gold nanoparticles, increase siRNA stability, specificity, and delivery efficiency, highlighting their immense potential in ELAVL1-targeted therapy. Nanotechnology offers unique advantages in enhancing drug efficacy and enabling an early diagnosis (80). However, several challenges remain: a. Toxicity and safety—Nanomaterials may trigger adverse effects, including inflammation, oxidative stress, and cytotoxicity, due to their rapid systemic distribution and potential accumulation in tissues such as the lungs (102). Metal nanoparticles, for example, are known to induce oxidative stress *in vivo (* 103). Immune responses to nanocarriers recognized as foreign substances can lead to immune-related complications (104). b. Efficient targeting—Despite ligand modifications for improved targeting, ensuring precise recognition and binding to specific cells remains an optimization challenge (105). Furthermore, the need to traverse multiple biological barriers during systemic delivery significantly affects siRNA delivery efficiency. c. siRNA stability and release—siRNAs are prone to nuclease degradation, reducing their stability and efficacy (92). In particular, immune recognition remains a nontrivial concern, as repeated exposure to nanomaterials may lead to innate immune activation, cytokine release, or even complement activation. Strategies such as PEGylation, biodegradable polymers, or use of biomimetic materials (e.g., exosomes) may help mitigate these effects. Additionally, chronic accumulation of non-degradable materials in organs such as liver or spleen poses potential risks that must be addressed through pharmacokinetic and toxicological studies. Nanocarriers must provide robust protection while enabling effective siRNA release under specific environmental conditions, such as pH, temperature, or enzymatic activity. d. Long-term safety—The long-term biocompatibility and potential off-target effects of nanocarriers on nontarget cells remain poorly understood, potentially impacting patient health (92). In conclusion, while nanomedicine offers transformative potential, their significant challenges require further exploration and optimization. Through advancements in nanomaterial design and fabrication, nanodelivery systems are poised to achieve substantial breakthroughs in drug delivery and gene therapy.

## 5 Conclusions

In summary, ELAVL1-targeted therapy using nanodelivery systems represents a promising new approach for the treatment of prostate cancer. Silencing ELAVL1 expression effectively inhibits the proliferation, invasion, and metastasis of prostate cancer cells. The use of nanodelivery systems increases the targeting and stability of the drug, allowing for precise drug delivery and improved antitumor effects. However, challenges remain in the clinical application of nanodelivery systems, including issues related to *in vivo* toxicity, safety, and manufacturing processes. Future research should focus on further exploring the safety and efficacy of these systems, as well as pursuing breakthroughs in personalized therapy, combination treatments, and overcoming drug resistance. In parallel, more rigorous *in vivo* studies and head-to-head comparisons with approved delivery platforms will be critical in establishing the translational relevance of ELAVL1-specific nanocarriers. Addressing long-term safety, immunogenicity, and manufacturing hurdles will be key steps toward clinical application.
